# Supplementary material for: A deep tensor-based approach for automatic depression recognition from speech utterances
Source: PLoS One. 2022 Aug 11;17(8):e0272659. doi: 10.1371/journal.pone.0272659 (PMC9371305; doi:10.1371/journal.pone.0272659)
Supplement: Attachment — Submitted filename: Response to Reviewers.pdf [file pone.0272659.s001.pdf]

I. Comments from reviewer 1

A. Comment

Please discuss the work of U of Michigan team on PRIORI which uses pitch to predict mood of patients with bipolar disorder. Mccinis, Khorram, and others are among the authors. Please compare your work with theirs and let us know how your work has enhanced their work.

Reply: We like to thank the reviewer for the suggestion. We have cited the manuscript and have described the work in the introduction section. The studies presented in ”The PRIORI Emotion Dataset: Linking Mood to Emotion Detected In-the-Wild” investigated emotion in speech as an intermediary feature for monitoring Bipolar Disorder over depressed and manic states of an individual. The authors proposed a new dataset collected from smartphone speech recordings and assessed several parameters such as PCC, CCC, RMSE etc to prove the robustnes of the feature and deep learning methods in identifying emotional activation and valence.

In contrast to the identified reference, we have examined depression recognition in an individual based on his/her speech utterances. The proposed architecture uniquely captures shared features across several utterances of a speaker spread temporally across the interview conversation. The emotion aspect as suggested by the reviewer is very interesting and is part of our future work in extending this study.

B. Comment

Please add the n to all your tables.

Reply: Thank you for the suggestion. However, we are not clear regarding the expectation of the reviewer in this comment. We have tried to conform to PLOS ONE Journal formatting guidelines.

C. Comment

Quality of figures are not acceptable. These figures are hard to follow.

Reply: We deeply regret the inconvenience in understanding the figures in the current form. To incorporate the reviewer's suggestion, we have added a brief description of the components of the figure in the caption to highlight the information flow along various components. We

have updated the figures to represent the end-to-end process starting from speech signal to label generation.

D. Comment

Too many abbreviations. Please reduce the use of abbreviations to those very nessasary.

Reply: As per the suggestion, to enhance the readability we have reduced the use of abbreviations throughout the paper.

E. Comment

PHQ8 and PHQ 9 differ, and their results should not be combined. Why not limiting the results to 8 items.

Reply: We agree with the observation of the reviewer. The DAIC-WoZ Dataset is annotated for depression using the PHQ-8 scale. Hence we have omitted any mention of PHQ-9 present in the manuscript.

F. Comment

If PHQ is used, why do you discuss and cite Hamilton measure too?

Reply:

Thank you, we had also deliberated on this aspect. Hamilton measure was included in the literature survey of the study to make the manuscript inclusive of background information for readers with non-clinical background. Accordingly, the primary intention of mentioning Hamilton scale was to inform the readers about several techniques used by the clinicians to generate labels for the mental health data.

G. Comment

We need tables that describe the participants.

Reply:

Thank you for the suggestion. We have added a table that summarizes the composition of the dataset with respect to participants according to the gender. However, certain identifying

details are not available in the Daic-Woz Dataset provided by the AVEC 2016 Challenge and the released baseline manuscript contained limited information about the participants.

H. Comment

Show us correlation between all variables. (Pearson r)

Reply:

Thank you for the suggestion. As we have used a deep learning model based on tensor factorization, the input to the method is 3D Tensors, which are stack of 2D Mel-spectrogram tensors along the third dimension. As such Pearson r coefficient of the input tensor is not defined. Moreover, since the stack of deep learning layers learn abstract information in the hidden layers, which are unknown in general, it is infeasible to provide correlation of variables in this particular proposed method.

I. Comment

Please use MDD for major depression. DOes not need the letter ”S”.

Reply: Thank you for the suggestion. We have made the suggested changes.

II. Comments from reviewer 2

A. Comment

The title of the article says depression recognition so the authors are only specific parameters based patterns generated to Recognition or authors may look the contextually fit then instead recognition detection is more suitable.? Justify with your answer.

Reply:. We thank the reviewer for the question. As suggested , we have replaced " detection" with "recognition" in line with the standards used in similar research papers such as -

1. L. Yang, D. Jiang, W. Han and H. Sahli, "DCNN and DNN based multi-modal depression recognition," 2017 Seventh International Conference on Affective Computing and Intelligent Interaction (ACII), 2017, pp. 484-489, doi: 10.1109/ACII.2017.8273643.

2.Hongying Meng, Di Huang, Heng Wang, Hongyu Yang, Mohammed AI-Shuraifi, and Yunhong Wang. 2013. Depression recognition based on dynamic facial and vocal expression features using partial least square regression. In *Proceedings of the 3rd ACM international workshop on Audio/visual emotion challenge* (*AVEC '13*). Association for Computing Machinery, New York, NY, USA, 21–30. https://doi.org/10.1145/2512530.2512532

B. Comment

The Word GIVE must be justified or reference adds for abbreviation if taken from research sources. Give the significant reasons for capitalization of the word

Reply:. Thank you for pointing out this. We have removed the word "GIVE" as it was not adding any extra information.

C. Comment

In page 1 line no. 6 of Introduction section Major Depression Disorder(MSDD) and Multi system depressive disorder must be given proper relation and both abbreviations cannot be interchangeable hence look into these point and justify your comments.

Reply: We thank the reviewer for pointing out this mistake. We have made the necessary correction and removed" Multisystem Depressive Disorder " as this was out of context here.

D. Comment

In Line No.10 page 2 the numbers added with the symbols of percentage and again word used so redundancy in information may be corrected. Ex: 80% percentage is not correct 80 percent or 80% along correct. Kindly justify your action on this modification

Reply: We thank the reviewer for pointing out this mistake. We have corrected as per the suggestion and only kept ”%” symbol and removed the word ”percent” to reduce redundant information.

E. Comment

Line 18 of page 2 “P.H.Q” abbreviated word usability in the entire document must be consistent.

Reply: We have modified the abbreviation according to the suggestion of the reviewer

F. Comment

Differentiation of the depression detection and depression recognition must be distinguished while using the work in the article. The usability of the word must be maintained in a consistent manner.

Reply: We thank the reviewer for this valuable comment. As per the suggestion of the reviewer, we have maintained uniformness across the paper by replacing ”detection” with ”recognition” as per the standards in the existing literature which utilizes deep learning in depression recognition.

G. Comment

Appreciate the authors with more grammatically correct and with simple sentence breaks made throughout the article in a consistent manner.

Reply: We thank the reviewer for raising this concern. We have tried to simplify sentences and improve grammar wherever possible in the paper.

H. Comment

Authors can justify properly the use of Abbreviations separated full stops and some places without them. Entire articles use a uniform process.

Reply: We thank the reviewer for pointing out this mistake. As per the suggestion, we have made the abbreviations uniform across the article.

I. Comment

Page No. 8 Section 3.3.2 given equations need to be numbered.

Reply: Thank you for the suggestion. We have added equation numbers for the same.

J. Comment

Page no. 9 and content in Table 1. Captions of the Row must be added to the percentage symbols as the accuracy is measured with ratios.

Reply: We have made the necessary changes as per the reviewer's suggestion.

K. Comment

Authors suggested rewriting the Abstract and Conclusions with simple sentences by appropriate breaks and conveying the authors view properly.

Reply: Thank you for the suggestion. We have tried to incorporate the reviewer's suggestion.

L. Comment

The technical concepts are good and explanation flow is appreciable.

Reply: We thank the reviewer for his kind appreciation.

III. Comments from the editor

A. Comment

In the related work, need to include more recently published work with explanation of what is the role of the deep learning research in depression analysis?

Reply:. We thank the editor for this suggestion. We have added reference to the more recent work as well as emphasized on the importance of deep learning for mental health diagnosis.

B. Comment

I suggest to more detailed explanation for Evaluation based on framework for automated depression classification and the compression with others recent work.

Reply:. We thank the editor for the suggestion. We have explained the evaluation procedure in detail in section 3.4 along with details on the hyperparameter values used. Also, the comparison with other recent work is limited to only those studies which have used accuracy and F1 score as metric and evaluated on test partition . Most of the other recent study has used regression metric and validation partition for evaluation, which is not included in our table of comparison.

C. Comment

Please explain in briefly and move their explanation in a paragraph explaining what each table and figures refer to.

Reply:. We thank the editor for the suggestion. We have added details about the information flow across various components in the diagram in the captions .

D. Comment

It will be helpful to have a few lines explaining what is inside the features chosen and why a particular fusion method is suitable for the particular depression analysis.

Reply:. We thank the editor for this suggestion. We have added a subsection titled " Discussion" which briefly explains the choice of mel-spectrograms as speech feature and what information 3D TFNN based MIL architecture tries to capture in its various layers.

REFERENCES
